# Prevalence of Asthma and Allergic Rhinitis and Their Association With Chronotype Among Adolescents in Ernakulam District: A School-Based Cross-Sectional Study

**DOI:** 10.7759/cureus.89928

**Published:** 2025-08-12

**Authors:** Parvathi Sureshkumar, Sreelakshmi Mohandas, Brilly M Rose, Aswathy Sreedevi

**Affiliations:** 1 Community Medicine, Amrita Institute of Medical Sciences, Amrita Vishwa Vidyapeetham, Ernakulam, IND

**Keywords:** adolescents, allergic rhinitis, asthma, chronotype, circadian preference, ernakulam, india, school-based

## Abstract

Background and objectives

Asthma and allergic rhinitis are common non-communicable diseases that cause chronic respiratory issues, poor sleep, and reduced academic performance among adolescents. Hence the objective of this study was to (1) estimate the prevalence of asthma and allergic rhinitis and (2) to determine its association with chronotype among adolescents in Ernakulam district, Kerala.

Methods

A school-based cross-sectional study was conducted between February and December, 2024, among adolescents aged 13 to 14 years from a selected school in urban Ernakulam using convenience sampling. The minimum calculated sample size with 95% confidence and an absolute precision of 5 was 303. Data were collected using the International Study of Asthma and Allergies in Childhood (ISAAC) questionnaire and the Ultra-Short Version of the Munich Chronotype Questionnaire. Logistic regression was done to identify the independent predictors for asthma and allergic rhinitis and results were reported as adjusted odds ratios (aOR) with 95% confidence intervals.

Results

Among the 313 participants interviewed, the prevalence of asthma was 15% (95% CI: 10.9-19.4), and allergic rhinitis was 24% (95% CI: 19.4-29.2). Most participants were of evening chronotype, 67% (95% CI: 61.8-72.6), followed by intermediate chronotype, 27% (95% CI: 22.1-32.1). The independent predictors for asthma were found to be morning chronotype [aOR:7.53 (2.4-23.4)] and household tobacco use [aOR: 5.21 (2.4-11.0)]. While the different chronotypes did not show any association with allergic rhinitis (p = 0.62), family history of asthma/allergy [aOR: 3.84 (2.0-7.2)] and larger family size [aOR: 1.89 (1.0-3.3)] were found to be independent predictors for allergic rhinitis.

Conclusion

The prevalence of asthma and allergic rhinitis was found to be high in our survey, with environmental factors and family history playing key roles. Asthma is linked to chronotype, while allergic rhinitis is not. It is recommended to implement preventive measures focusing on modifiable risk factors like tobacco use and the early detection of high-risk chronotypes.

## Introduction

Asthma and allergic rhinitis (AR) are among the most prevalent chronic respiratory disorders globally, exerting a substantial burden on the health and well-being of all. According to the World Health Organization (WHO), asthma currently affects approximately 300 million individuals worldwide, a figure projected to rise to 400 million in 2025 [1]. It is estimated to cause around 250,000 deaths annually, with a disproportionately high burden in low- and middle-income countries [1]. In the United States, asthma contributes to the loss of over 10 million school days annually and ranks as one of the leading causes of paediatric hospitalization [2].

AR, a highly prevalent allergic airway disease, affects 10%-40% adults and 2%-25% children globally, with rising trends observed in urbanized and industrialized regions [3]. In South-East Asia, prevalence of AR ranges from 6% to 22%, with significant geographic variation influenced by environmental exposures such as indoor and outdoor air pollution [4,5].

Beyond their high prevalence, asthma and AR significantly compromise quality of life and daily functioning. Children and adolescents with asthma and AR may experience frequent episodes of wheezing, coughing, breathlessness, chest tightness, hyperresponsiveness, nasal congestion, sneezing, rhinorrhoea, and itching which can disrupt sleep, impair concentration, and negatively impact academic performance and social interactions. These symptoms are frequently exacerbated at night, leading to sleep disturbances, increased fatigue, reduced physical activity, and school absenteeism [6,7].

An emerging area of interest in paediatric respiratory health is the influence of circadian rhythms on disease expression and severity. Circadian rhythms, governed by endogenous biological clocks, orchestrate daily physiological and behavioural processes, including immune function and airway tone [8]. During adolescence, shifts in sleep-wake cycles and a natural delay in circadian phase, referred to as evening chronotype, can lead to misalignment between internal biological rhythms and external social schedules [9]. Chronotype, also known as circadian preference, refers to an individual’s preferred timing of sleep and activity within a 24-hour period and is a behavioral manifestation of the circadian system. It is commonly categorized into morning, intermediate, and evening type [10]. Morning chronotypes tend to wake up and be most alert early in the day, often going to bed and rising earlier than others. Evening chronotypes prefer later bedtimes and wake times, with peak alertness and activity occurring in the evening hours. Intermediate chronotypes fall between these two extremes and do not show a strong preference for early or late activity patterns.

Studies have demonstrated that asthma symptoms commonly worsen during the night, attributed to circadian variation in bronchomotor tone and inflammatory mediators. Moreover, misalignment of sleep timing due to evening chronotype or irregular schedules may exacerbate circadian disruption, impacting immune responses and increasing susceptibility to airway inflammation [11]. Disruption in melatonin secretion, caused by exposure to artificial light at night, irregular sleep patterns, or chronotype mismatch, has also been linked to a higher risk of asthma and allergic diseases [12].

In this context, the objective of the present study was to estimate the prevalence of asthma and allergic rhinitis and to determine their association with chronotype among adolescents in Ernakulam district of Kerala.

## Materials and methods

A cross-sectional study was conducted in a selected (convenience sampling) school in Ernakulam district between February - December 2024, targeting adolescents aged 13-14 years. This age group was chosen because it represents early adolescence, a developmental period when circadian phase delay becomes biologically prominent, making it an appropriate stage to study chronotype-related factors. Additionally, the International Study of Asthma and Allergies in Childhood (ISAAC) Phase III recommends this age group for asthma prevalence studies, ensuring comparability with both national and international literature. The school was selected based on its urban location and willingness of the school administration to participate. The minimum calculated sample size was 303, with a reported prevalence of 25.33% from urban Jaipur, with 95% confidence and an absolute error of 5 [13].

Participants with recent upper respiratory infections (within the past week), diagnosed psychiatric conditions affecting sleep, and current use of medications influencing circadian rhythms, such as melatonin or stimulants were excluded from the study.

Data were collected using a semi-structured, three-part questionnaire: (a) Details regarding the sociodemographic profile, covering household environment, cooking practices, and pet ownership; (b) the ISAAC questionnaire [14], which captured physician-diagnosed asthma and allergic rhinitis and (c) the Ultra-Short Munich Chronotype Questionnaire (µMCTQ), which assesses the chronotype by recording separate sleep and wake times for school days and free days. Mid-sleep on free days (MSF) was used as the primary measure of determining chronotype. MSF was corrected for sleep debt accumulated during school days to obtain the sleep-corrected mid-sleep on free days (MSFsc). MSFsc is used to determine the mid sleep point based on which the chronotype is categorized into three. The mid sleep point is calculated using the formula: MSF − 0.5 × (Sleep duration on free days − Sleep duration on school days). Participants were then classified into the three chronotypes: morning chronotype (MSFsc <3:00 AM), intermediate chronotype (MSFsc between 3:00-5:00 AM), or evening chronotype (MSFsc >5:00 AM) [15].

Prior to data collection, written informed consent from the parents and assent from the adolescents were obtained. The questionnaire was paper-based and administered in English by the researcher in person at school to ensure clarity. Data were entered in Microsoft Excel (Microsoft, Redmond, WA, USA) and analysed using Jamovi 2.4 software (https://www.jamovi.org) and SPSS Statistics for Windows, Version 21.0 (IBM Corp., Armonk, NY, USA). Descriptive statistics are summarized as continuous (means) and categorical (frequencies with percentages) variables. Chi-square tests for association assessed the associations between categorical variables. Variables with p value < 0.2 in bivariate analysis were included in multivariable logistic regression to identify independent predictors of asthma and allergic rhinitis. Regression outcomes were reported as adjusted odds ratios (aOR) with 95% confidence intervals (CI). Multicollinearity was assessed using Variance Inflation Factor (VIF), where a VIF > 5 was considered indicative of multicollinearity.

The study received approval from the Institutional Ethics Committee (Letter No.: ECASM-AIMS-2024-617, Date: 13-12-2024). Formal permission to reproduce the full µMCTQ [15] was obtained from SAGE Publications via RightsLink.

## Results

A total of 313 adolescents aged 13-14 years participated in the study. Of these, 52% (n=163) were 14 years old. The gender distribution was fairly balanced, with 51% (n=160) being males. The majority of participants, 70% (n=219), resided in urban areas. In terms of socioeconomic status, 92% (n=288) belonged to Above Poverty Line (APL) households. Family type distribution showed that 53% (n=165) were from nuclear families, 13% (n=41) from joint families, and 34% (n=107) from three-generation families. A majority, 61% (n=191) of the participants, came from families with fewer than five members. Most households, 98% (n=307), used liquefied petroleum gas (LPG) as the primary cooking fuel, while others reported using electricity and various biomass fuels such as firewood, charcoal, and kerosene. A family history of asthma or allergy was reported by 61% (n=191) of participants; 36% (n=113) had pets at home, and 16% (n=51) reported exposure to tobacco use within the household (Table 1). The tables with detailed sociodemographic distribution of participants by asthma status and by allergic rhinitis status are provided in Appendices 1 and 2, respectively.

**Table 1:** Participant Sociodemographic Characteristics (n=313) Values are expressed as frequency (n) and percentage (%) APL: above poverty line, BPL: below poverty line, LPG: liquefied petroleum gas

Variable	Categories	Frequency n (%)
Age (in years)	13	150 (47.9)
	14	163 (52.1)
Gender	Male	160 (51.1)
	Female	153 (48.9)
Location of Residence	Urban	220 (70.3)
	Rural	93 (29.7)
Socioeconomic Status	APL	288 (92.01)
	BPL	25 (7.99)
Type of Family	Nuclear	165 (52.7)
	Joint	42 (13.4)
	Three Generation	106 (33.9)
Family Size	≤ 5	191 (61.0)
	> 5	122 (39.0)
Cooking Fuel Use *(multiple options)*	LPG	306
	Electricity	88
	Firewood	38
	Charcoal	1
	Kerosene	5
	Solar Energy	7
Family History of Asthma/Allergy	Yes	190 (60.7)
	No	123 (39.3)
Pets at Home	Yes	111 (35.5)
	No	202 (64.5)
Household Tobacco Use	Yes	50 (16)
	No	263 (84)

Physician-diagnosed asthma was reported in 15% (n=46) of participants (95% CI: 10.9-19.4), and the prevalence of allergic rhinitis was 24% (n=75) (95% CI: 19.4-29.2). Among the 313 participants, 6% (n=18) (95% CI: 3.4-9.2) had a morning chronotype, 27% (n=84) (95% CI: 22.1-32.1) had an intermediate chronotype, and 67% (n=211) (95% CI: 61.8-72.6) exhibited an evening chronotype (Table 2).

**Table 2:** Prevalence of asthma, allergic rhinitis and chronotype (n=313) Values are presented as frequency (n) and percentage (%). Asthma and allergic rhinitis were physician-diagnosed.

	Category	Frequency n (%)
Asthma prevalence	Present	46 (14.7)
	Absent	267 (85.3)
Allergic rhinitis prevalence	Present	75 (24)
	Absent	238 (76)
Chronotype	Morning	18 (5.8)
	Intermediate	84 (26.8)
	Evening	211 (67.4)

A statistically significant association with asthma was established for family history of asthma/allergy [OR: 2.64 (1.2-5.5)], household tobacco use [OR: 4.73 (2.3-9.3)], and chronotype [OR: 5.95 (2.1-16.5)] in the bivariate analysis. The significant predictors for asthma in the multivariable model were morning chronotype [aOR: 7.53 (2.4-23.4)] and the presence of household tobacco use [aOR: 5.21 (2.4-11.0)] (NR² = 0.217. All predictor variables had VIF values < 5, confirming no evidence of multicollinearity) (Table 3).

**Table 3:** Factors associated with asthma (n=313) Values are expressed as frequency (n), percentage (%), odds ratio (OR), and adjusted odds ratio (aOR) with 95% confidence intervals. Chi-square test (χ²) was used for bivariate comparisons. p-values <0.05 were considered statistically significant.

Variable	Present n (%)	Absent n (%)	p-value	χ² value	OR (95%CI)	aOR (95%CI)	p -value
								Location							
Urban	27 (12.3)	193 (87.7)	0.063	3.47	0.54 (0.28-1.04)	1.90 (0.93-3.86)	0.075
Rural	19 (20.4)	74 (79.6)			Ref	1	
Family history of asthma/allergy							
Yes	36 (18.9)	154 (81.1)	<0.001	6.97	2.64 (1.26-5.54)	2.09 (0.96-4.57)	0.063
No	10 (8.1)	113 (91.9)			Ref	1	
Household tobacco use							
Yes	18 (36.0)	32 (64.0)	<0.001	3.29	4.73 (2.38-9.38)	5.21(2.45-11.09)	<0.001
No	28 (10.6)	235 (89.4)			Ref	1	
Chronotype							
Morning	8 (44.4)	10 (55.6)	<0.001	14.1	5.95 (2.14-16.49)	7.53 (2.42-23.39)	<0.001
Intermediate	13 (15.5)	71 (84.5)			1.36 (0.66-2.80)	1.57 (0.72-3.42)	0.251
Evening	25 (11.8)	186 (88.2)			Ref	1	

Allergic rhinitis was significantly associated with a family history of asthma or allergy [OR: 3.68 (1.9-6.9)] and with larger family size (more than four members) [OR: 1.74 (1.0-2.9)] in bivariate analysis. Household tobacco use [OR: 1.84 (0.9-3.4)] and chronotype did not show a significant association with allergic rhinitis (p = 0.619). In the multivariable logistic regression model, family history of asthma or allergy [aOR: 3.84 (2.0-7.2)] and larger family size [aOR: 1.89 (1.0-3.3)] remained as significant independent predictors for allergic rhinitis (NR² = 0.126. All predictor variables had VIF values < 5, confirming no evidence of multicollinearity) (Table 4).

**Table 4:** Factors associated with allergic rhinitis (n=313) Values are expressed as frequency (n), percentage (%), odds ratio (OR), and adjusted odds ratio (aOR) with 95% confidence intervals. Chi-square test (χ² )was used for bivariate comparisons. p-values <0.05 were considered statistically significant.

Variable	Present n (%)	Absent n (%)	p-value	χ² value	OR (95%CI)	aOR (95%CI)	p -value
								Family history of asthma/allergy							
Yes	61 (32.1)	129 (67.9)	<0.001	17.6	3.68 (1.95–6.94)	3.84 (2.02-7.28)	<0.001
No	14 (11.4)	109 (88.6)			Ref	1	
Family size							
≤4	53 (27.7%)	138 (72.3)	0.051	3.86	1.74 (1.00–2.98)	1.89 (1.06-3.37)	0.03
>4	22 (18.0%)	100 (82.0)			Ref	1	

## Discussion

Prevalence of asthma and allergic rhinitis

In this school-based cross-sectional study among adolescents aged 13-14 years, the prevalences of physician-diagnosed asthma and allergic rhinitis were 15% and 24%, respectively. These findings align with prior reports from various parts of India, such as Delhi [16], Andaman and Nicobar Islands, Daman and Diu [17], Chandigarh [18], Lakshadweep, Dadra and Nagar Haveli [19] and Puducherry [20], indicating a rising burden of allergic respiratory diseases in children and adolescents, particularly in urban environments with high exposure to indoor and outdoor pollutants.

Chronotype

In our study, morning chronotype was significantly associated with higher odds of physician-diagnosed asthma. This finding contrasts with earlier studies, which more commonly report an association between evening chronotype and increased asthma risk, likely due to a mismatch between their natural sleep-wake rhythm and externally imposed schedules such as early school timings, resulting in sleep deprivation and circadian misalignment [7,9]. 

Circadian misalignment, which refers to a discrepancy between an individual’s biological rhythms and social schedules, has been shown to exacerbate respiratory symptoms and may be influenced by factors such as early school start times, excessive evening screen use, and insufficient sleep duration. Marseglia et al. have described the role of melatonin in asthma pathophysiology, suggesting that any disruption in circadian timing may contribute to airway inflammation [11]. Although these variables were not directly measured in our study, they represent important potential mediators that warrant further investigation.

The unexpected association with morning chronotype may be explained by several factors. First, the number of students with morning chronotype was small (n=18), which may have inflated the odds ratio. Second, reverse causation is possible; adolescents with asthma might adopt earlier sleep schedules to manage symptoms or due to parental influence. Third, misclassification bias cannot be ruled out because chronotype was self-reported and may have been affected by school-imposed routines. These findings, while intriguing, should be interpreted cautiously and validated in larger, multi-centre studies using objective measures of circadian preference such as actigraphy.

The high prevalence of evening chronotype (67.4%) observed in our study is typically seen in adolescence but may also be amplified by factors like high rates of evening screen exposure, academic demands leading to late-night study habits, and urban lifestyle patterns, which could further delay sleep timing. Exploring these behavioural and environmental factors could provide additional insight into chronotype distribution and its health implications among adolescents.

No significant association was observed between chronotype and allergic rhinitis, aligning with previous reports suggesting that allergic rhinitis is more likely driven by genetic and environmental factors than by chronobiological influences [21]. Future research should incorporate mediating factors such as screen use, school schedules, and sleep duration to clarify the pathways linking chronotype and asthma and to inform potential school-based interventions aimed at improving sleep hygiene and circadian alignment.

Household tobacco exposure

Household tobacco smoke exposure emerged as another key risk factor for asthma, with exposed adolescents having nearly sixfold higher odds of developing the condition. This is consistent with a study conducted by Aswathy S et al. in Ernakulam district, Kerala, which reported high levels of tobacco use and second-hand smoke exposure among high school students [22]. However, it is possible that adolescents may underreport household tobacco exposure due to social desirability bias, which could lead to an underestimation of this association. These findings reinforce the need for targeted awareness campaigns and smoking cessation interventions at the family level, particularly because children have little control over their home environment.

Family history of asthma or allergy

A family history of asthma or allergy was significantly associated with allergic rhinitis, underscoring the role of genetic and epigenetic predispositions in atopic conditions. Prior evidence suggests that children with one or both parents affected by asthma or allergies are at a substantially increased risk of developing similar conditions due to shared genetic polymorphisms [23]. This finding emphasizes the value of family-centered screening strategies, especially in primary care settings.

Family size

Larger family size (more than four members) was found to be a significant predictor of allergic rhinitis in our study. The hygiene hypothesis suggests that greater household size may protect against allergic diseases by increasing early-life microbial exposure. However, in urban Indian settings, characterized by reduced exposure to outdoor microbial diversity, increased indoor allergen load, and lifestyle changes such as higher use of cleaning products and reduced natural ventilation, this protective effect may be offset, potentially increasing the risk [24,25]. As our study did not explore these household-level exposures in depth, further research is needed to better understand this association 

Other factors

Notably, residence, socioeconomic status (SES) and pet exposure did not significantly influence asthma or allergic rhinitis prevalence in multivariable models. While pet exposure has been implicated in allergic sensitization in some contexts, the evidence remains mixed, and early-life exposure may in fact confer immunological tolerance [26].

Strengths and limitations

The use of validated instruments such as the ISAAC questionnaire and the Ultra-Short Munich Chronotype Questionnaire, as well as the application of multivariable analysis to adjust for potential confounders, are strengths of this research. 

The narrow age range (13-14 years) improves developmental homogeneity and aligns with ISAAC methodology but limits applicability to other adolescent age groups. Additionally, our initial hypothesis did not specifically focus on chronotype; therefore, the sample size calculation was based on estimating asthma prevalence rather than modelling the association between specific chronotypes and asthma. Consequently, causal modelling approaches were not employed. Furthermore, the use of self-reported data for asthma, allergic rhinitis, and sleep timing may be subject to recall or social desirability bias, particularly in adolescents. 

School-specific environmental or socioeconomic factors may have influenced the prevalence of asthma and allergic rhinitis as well as chronotype distribution.

## Conclusions

This study highlights the considerable burden of asthma and allergic rhinitis among adolescents in an urban school in Ernakulam, with evidence suggesting links to chronobiological patterns, environmental exposures, and family history. While our findings are exploratory, they indicate the potential value of sleep hygiene education and chronotype awareness programs in schools. However, these strategies should be viewed as recommendations for further investigation rather than immediate implementation, given the study’s limited generalizability. Targeted health education for students and families, particularly regarding modifiable risk factors such as household tobacco exposure, is also warranted. Future research should include larger, multi-site studies across Kerala and other regions of India, using longitudinal designs and objective measures of sleep and circadian rhythms to better establish causality. With Kerala’s high literacy and health awareness, such studies could provide the foundation for evidence-based school interventions in the future.

## Appendices

Appendix 1 

**Table 5:** Sociodemographic characteristics of participants with asthma (n=46) Values are expressed as frequency (n) and percentage (%)

Variable	Asthma n%
Age	
13 years	24 (16.0%)
14 years	22 (13.5%)
Gender	
Male	23 (14.4%)
Female	23 (15.0%)
Residence	
Urban	27 (12.3%)
Rural	19 (20.4%)
Socioeconomic status	
APL	41 (14.2%)
BPL	5 (20.0%)
Family type	
Nuclear	27 (16.4%)
Joint	9 (21.4%)
Three generation	10 (9.4%)
Family size	
≤4	28 (14.7%)
>4	18 (14.8%)
Family history of asthma/allergy	
Yes	36 (18.9%)
No	10 (8.1%)
Pets at Home	
Yes	19 (17.1%)
No	27 (13.4%)
Household tobacco use	
Yes	18 (36.0%)
No	28 (10.6%)
Chronotype	
Morning	8 (44.4%)
Intermediate	13 (15.5%)
Evening	25 (11.8%)

Appendix 2 

**Table 6:** Sociodemographic characteristics of participants with allergic rhinitis (n=75) Values are expressed as frequency (n) and percentage (%)

Variable	Allergic Rhinitis n%
Age	
13 years	41 (27.3%)
14 years	34 (20.9%)
Gender	
Male	37 (23.1%)
Female	38 (24.8%)
Residence	
Urban	51 (23.2%)
Rural	24 (25.8%)
Socioeconomic status	
APL	70 (24.3%)
BPL	5 (20.0%)
Family type	
Nuclear	45 (27.3%)
Joint	9 (21.4%)
Three generation	21 (19.8%)
Family size	
≤4	53 (27.7%)
>4	22 (18.0%)
Family history of asthma/allergy	
Yes	61 (32.1%)
No	14 (11.4%)
Pets at Home	
Yes	27 (24.3%)
No	48 (23.8%)
Household tobacco use	
Yes	17 (34.0%)
No	58 (22.1%)
Chronotype	
Morning	6 (33%)
Intermediate	19 (22.6%)
Evening	50 (23.7%)
